# Dynamic Expression of m^6^A Regulators During Multiple Human Tissue Development and Cancers

**DOI:** 10.3389/fcell.2020.629030

**Published:** 2021-01-26

**Authors:** Ya Zhang, Sicong Xu, Gang Xu, Yueying Gao, Si Li, Ke Zhang, Zhanyu Tian, Jing Guo, Xia Li, Juan Xu, Yongsheng Li

**Affiliations:** ^1^Key Laboratory of Tropical Translational Medicine of Ministry of Education, Hainan Medical University, Haikou, China; ^2^College of Biomedical Information and Engineering, Hainan Medical University, Haikou, China

**Keywords:** m^6^A regulators, tissue development, cancer, gene experession, survival

## Abstract

N6-methyladenosine (m^6^A) plays critical roles in human development and cancer progression. However, our knowledge regarding the dynamic expression of m^6^A regulators during human tissue development is still lacking. Here, we comprehensively analyzed the dynamic expression alterations of m^6^A regulators during seven tissue development and eight cancer types. We found that m^6^A regulators globally exhibited decreased expression during development. In addition, IGF2BP1/2/3 (insulinlike growth factor 2 MRNA-binding protein 1/2/3) exhibited reverse expression pattern in cancer progression, suggesting an oncofetal reprogramming in cancer. The expressions of IGF2BP1/2/3 were regulated by genome alterations, particularly copy number amplification in cancer. Clinical association analysis revealed that higher expressions of IGF2BP1/2/3 were associated with worse survival of cancer patients. Finally, we found that genes significantly correlated with IGF2BP1/2/3 were significantly enriched in cancer hallmark-related pathways. In summary, dynamic expression analysis will guide both mechanistic and therapeutic roles of m^6^A regulators during tissue development and cancer progression.

## Introduction

N6-methyladenosine (m^6^A) is the most abundant RNA modification and has been shown to play important roles in development and cancer (Dominissini et al., 2012; Meyer et al., 2012). RNA methylation has been shown to be catalyzed by a multicomponent methyltransferase complex, including METTL13/14 and WTAP (Liu et al., 2014). RNA methylation has been found in thousands of coding and non-coding genes. However, we still lack knowledge about the functions of m^6^A regulators in development and cancer.

Numerous studies have shown that m^6^A regulators were widely perturbed in various types of cancer. METTL3 and IGF2BP2 (insulinlike growth factor 2 MRNA-binding protein 2) were overexpressed in colorectal cancer and promote the cancer progression (Li et al., 2019). WTAP was also shown to lead to suppression of ETS1 and contribute to the proliferation of liver cancer (Chen et al., 2019b). Xu et al. found that 19 m^6^A regulators were highly expressed in glioma tissues, and the expression of regulators was associated with prognoses and grade (Xu et al., 2020b). One recent study has revealed an essential role of YTHDF1 in the progression of hepatocellular carcinoma (Liu et al., 2020). RNA methyltransferase METTL3 has been found to facilitate colorectal cancer by activating m6A-GLUT1-mTORC1 axis and is a therapeutic target (Chen et al., 2020). Moreover, we recently performed comprehensive characterization of m^6^A regulators in 33 cancer types and found that there were widespread expression perturbation of m^6^A regulators in cancer (Li et al., 2019a). However, there has been no research that comprehensively explored the expression landscape of m^6^A regulators during human tissue development.

In this study, we performed a comprehensive evaluation of the expression of m^6^A regulators across multiple human tissues development and cancer. We found that the m^6^A regulators exhibited decreased expression after born. Moreover, m^6^A regulators were up-regulated in cancer, which suggests an oncofetal reprogramming mechanism in cancer. Moreover, we investigated the association between the expression of m^6^A regulators and patient survival. Our comprehensive analysis of the dynamic expression of m^6^A regulators provided novel insights into their function in development and cancer.

## Materials and Methods

### Transcriptome During Human Tissue Development

Gene expression data across human tissue development were downloaded from ArrayExpress with the accession code E-MTAB-6814 (Cardoso-Moreira et al., 2019). The samples were started from prenatal development at 4 weeks postconception (WPC) to 20 WPC. Moreover, postnatal samples were also sampled, including infants, toddlers, school, teenagers, and adults from each decade until 65 years of age. There were seven human tissues analyzed, including brain, cerebellum, heart, kidney, liver, ovary, and testis. In total, 297 human samples were sequenced by RNA-Seq. Gene expression levels were calculated as reads per kilobase of exon model per million mapped reads.

### Gene Expression in Human Cancers

We downloaded the gene expression across human cancer types from The Cancer Genome Atlas (TCGA) project (Cancer Genome Atlas Research et al., 2013). According to the tissues in human development, we considered eight cancer types, including kidney renal clear cell carcinoma (KIRC), kidney renal papillary cell carcinoma (KIRP), kidney chromophobe (KICH), brain lower grade glioma, glioblastoma multiforme, ovarian serous cystadenocarcinoma, liver hepatocellular carcinoma (LIHC), and testicular germ cell tumors. Gene expression levels were calculated as fragments per kilobase of exon model per million mapped fragments. Moreover, we downloaded the clinical information of patients, including the survival time, survival status from TCGA project.

To validate the expression of IGF2BPs regulators across cancer types, we collected gene expression data across ~7,400 samples representing 11 cancers. Gene expression data were collected from Gene Expression Omnibus. To minimize interplatform variation, only datasets generated from the Affymetrix Human Genome U133 Plus 2.0 Array were processed to develop the meta-dataset (Bin Lim et al., 2019). Each dataset was preprocessed with RMA normalization, merged, and batch effect–corrected via Combat method (Leek et al., 2012).

### Collection of m^6^A Regulators

The gene list of m^6^A regulators was obtained from one of our studies (Li et al., 2019b), which curated a catalog of 20 genes that function mainly as regulators of RNA methylation. In total, there were 11 readers, 7 writers, and 2 erasers. All these gene symbols were converted into Ensemble gene IDs and HGNC symbols based on annotation from GeneCards (https://www.genecards.org/).

### Differential Expression Analysis

To evaluate whether the expression of m^6^A regulators exhibits differential expression between normal and cancer, we used the Wilcoxon rank sum test to compare the expression levels. Here, only cancer types with more than five normal samples were analyzed. The gene expression of m^6^A regulators across normal or cancer samples was shown by box plots.

### Genome Alteration of m^6^A Regulators

To explore the genome alteration of m^6^A regulators, we used the cBioPortal tool (Gao et al., 2013), which provided a Web resource for exploring, visualizing, and analyzing multidimensional cancer genomic data. The gene symbols of m^6^A regulators were used as input, and TCGA pan-cancer datasets were used. The genome alteration frequency of m^6^A regulators was calculated and plotted as bar plot. Here, genetic mutations and copy number variation were considered.

### Survival Analysis

To explore whether the expression of m^6^A regulators was associated with patient survival, we divided all the patients into two groups based on the median expression of each m^6^A regulator. The log-rank test was used to test the difference survival rates between two groups. This process was performed by the survival package in R program (https://cran.r-project.org/web/packages/survival/index.html). The *p* < 0.05 was considered as significant. The odds ratios and 95% confidence levels were also calculated and shown by ggplot2 package in R program (Wickham, 2009).

### Function Prediction of Regulators

To predict the function of m^6^A regulators in human development and cancer, we first calculated the Spearman correlation coefficient (SPCC) between the expression of m^6^A regulators and other protein coding genes. All the genes were ranked by SPCC and subjected to preranked Gene Set Enrichment Analysis (GSEA) (Mootha et al., 2003; Subramanian et al., 2005). The cancer hallmark-related functions were considered (Liberzon et al., 2011, 2015). Gene sets with more than 5 genes and fewer than 1,500 genes were considered. We performed 1,000 times randomization. The normalized enrichment scores of GSEA were shown by “pheatmap” function in R program (https://cran.r-project.org/web/packages/pheatmap/index.html).

## Results

### Dynamic Expression of m^6^A Regulators During Tissue Development

**Figure 1:**
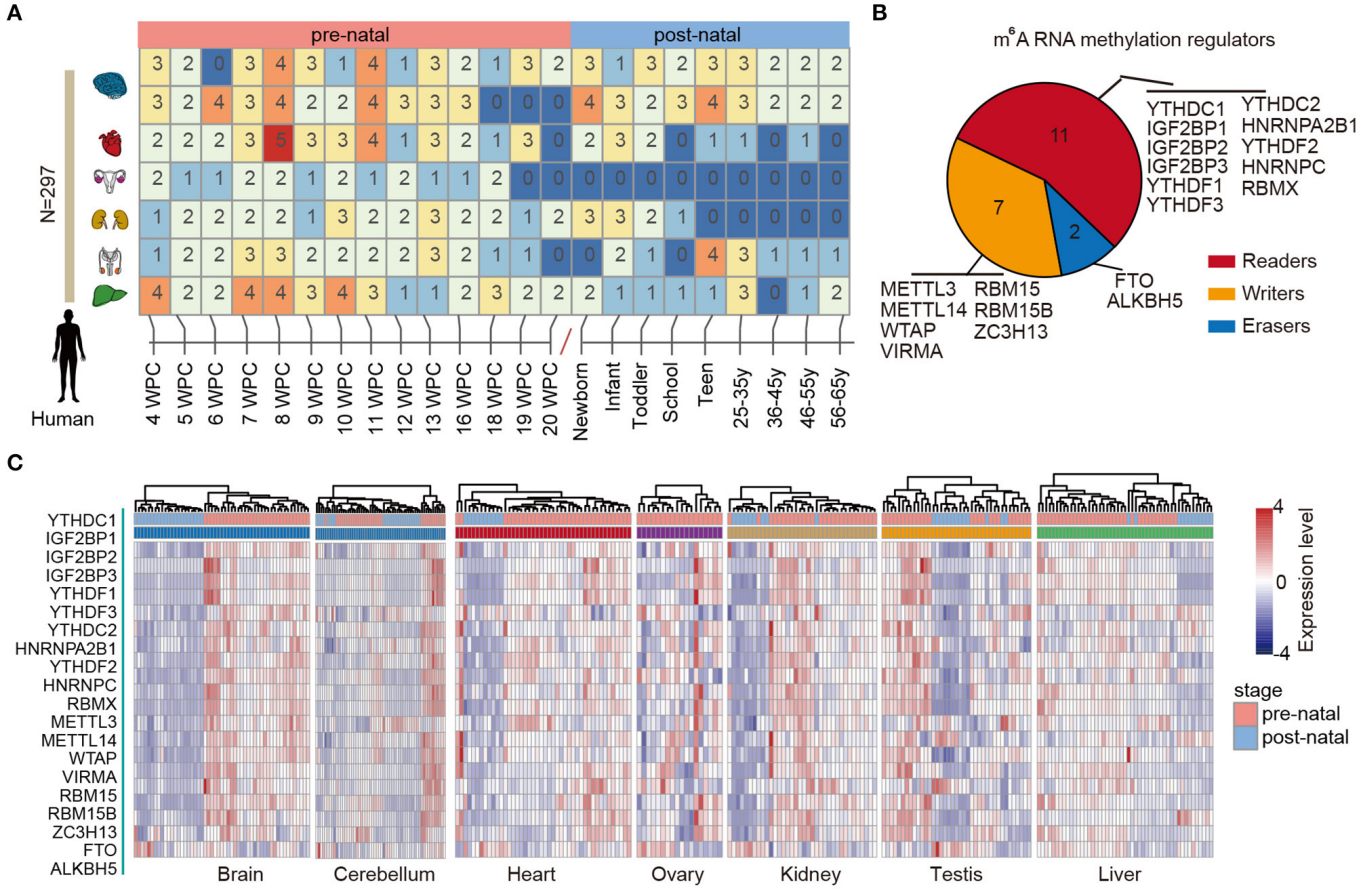
Dynamic expression of m^6^A regulators during tissue development. **(A)** Heat map showing the number of samples in different development stages of multiple tissues. **(B)** Pie chart for the number of m^6^A regulators, including 11 readers, 7 writers, and 2 erasers. **(C)** Heat maps showing the expression of m^6^A regulators in different tissues. The prenatal and postnatal samples were indicated with red and blue colors.

RNA methylation is the most abundant modification and play important roles in various types of biological processes, including tissue development and cancer. We first characterized the dynamic expression m^6^A regulators across multiple human tissue development (Figure 1A). In total, 297 samples in seven tissues were collected and sequenced by RNA-Seq. We first divided the samples into two main groups: prenatal vs. postnatal. Next, we collected 20 m^6^A regulators from literature. These regulators were classified into reads, writers, and erasers (Figure 1B).

Next, we clustered the samples in different tissues based on the expression of m^6^A regulators. We found that the prenatal and postnatal samples were clearly distinguished from each other (Figure 1C). Globally, the m^6^A regulators exhibited higher expression in prenatal samples than postnatal across all tissues. These results suggest that m^6^A exhibited decreased expression after birth. Particular, this trend was much clearer in brain and heart tissues (Figure 1C). Moreover, we found that ALKBH5 exhibited lower expression in prenatal samples. All these results suggest that there might be great changes of RNA methylation during tissue development.

### IGF2BP1/2/3 Exhibit High Expression in Prenatal Tissues

**Figure 2:**
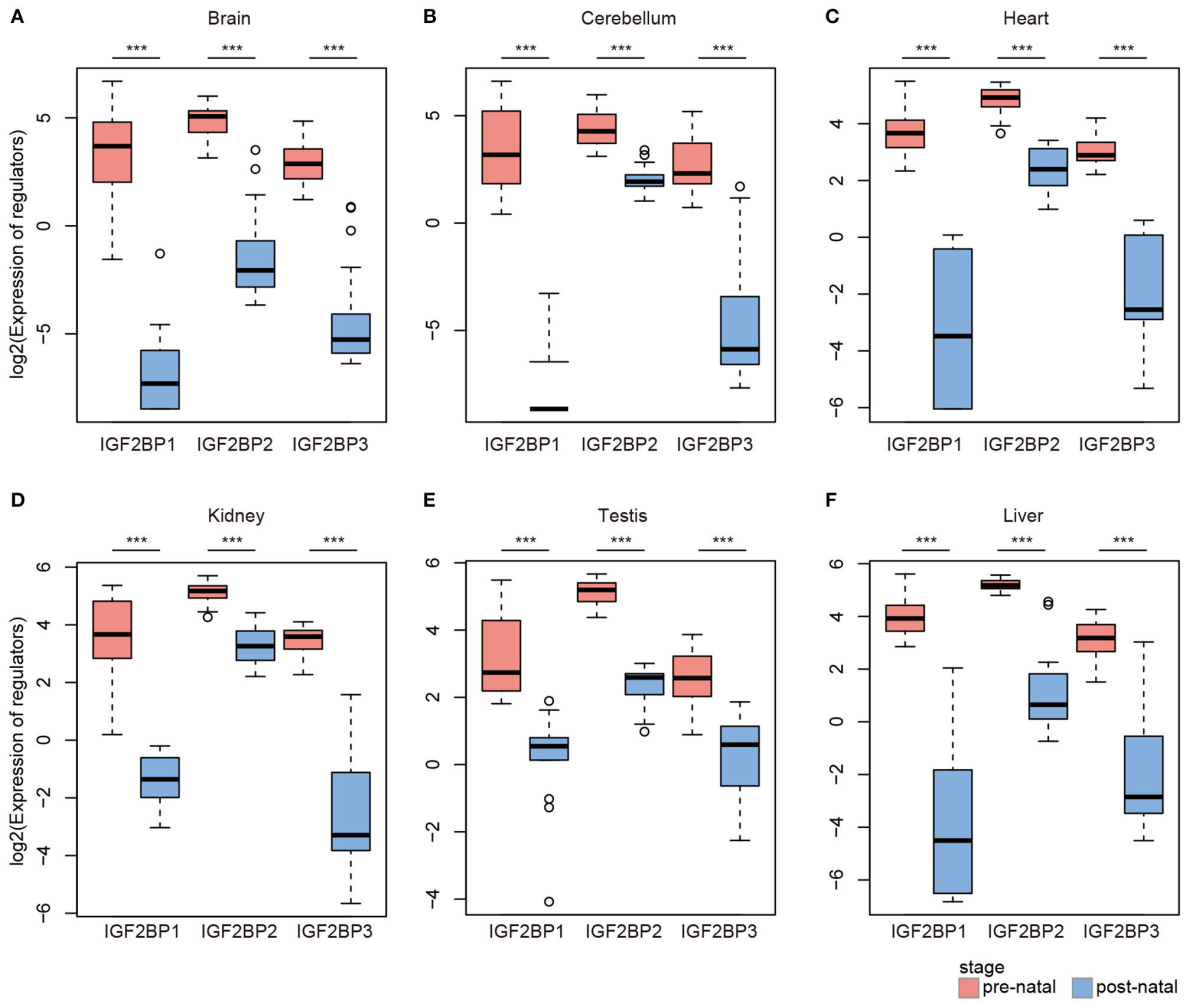
Box plots showing the expression of IGF2BP1/2/3 in prenatal and postnatal samples of different tissues. **(A)** Expression of IGF2BPs in brain tissues. **(B)** Expression of IGF2BPs in cerebellum tissues. **(C)** Expression of IGF2BPs in heart tissues. **(D)** Expression of IGF2BPs in kidney tissues. **(E)** Expression of IGF2BPs in testis tissues. **(F)** Expression of IGF2BPs in liver tissues. ***P < 0.001 for Wilcoxon rank sum test.

Based on global heat map of the expression of m6A regulators, we found that they exhibited higher expression in prenatal samples. Particularly, we revealed that three readers IGF2BP1, IGF2BP2, and IGF2BP3 showed higher expression in prenatal samples across all tissues. Next, we comprehensively compared the expression levels of three IGF2BPs across seven tissues. We found that all three genes exhibited significantly higher expression in prenatal samples in all tissues (Figure 2). IGF2BP1 overexpression had been found to cause fetal-like hemoglobin expression patterns in cultured human adult erythroblasts (de Vasconcellos et al., 2017). We found that IGF2BP1 were with significantly lower expression in postnatal samples in brain and cerebellum tissues. We next queried PubMed for exploring the function of IGF2BP1, IGF2BP2, and IGF2BP3 in tissue development. A number of studies have investigated their function in cancer; however, there were limited number of literature were retrieved for tissue development. Taken together, all these results suggest that IGF2BPs exhibit decreased expression pattern during development.

### Reverse Expression of IGF2BP1/2/3 in Cancer

**Figure 3:**
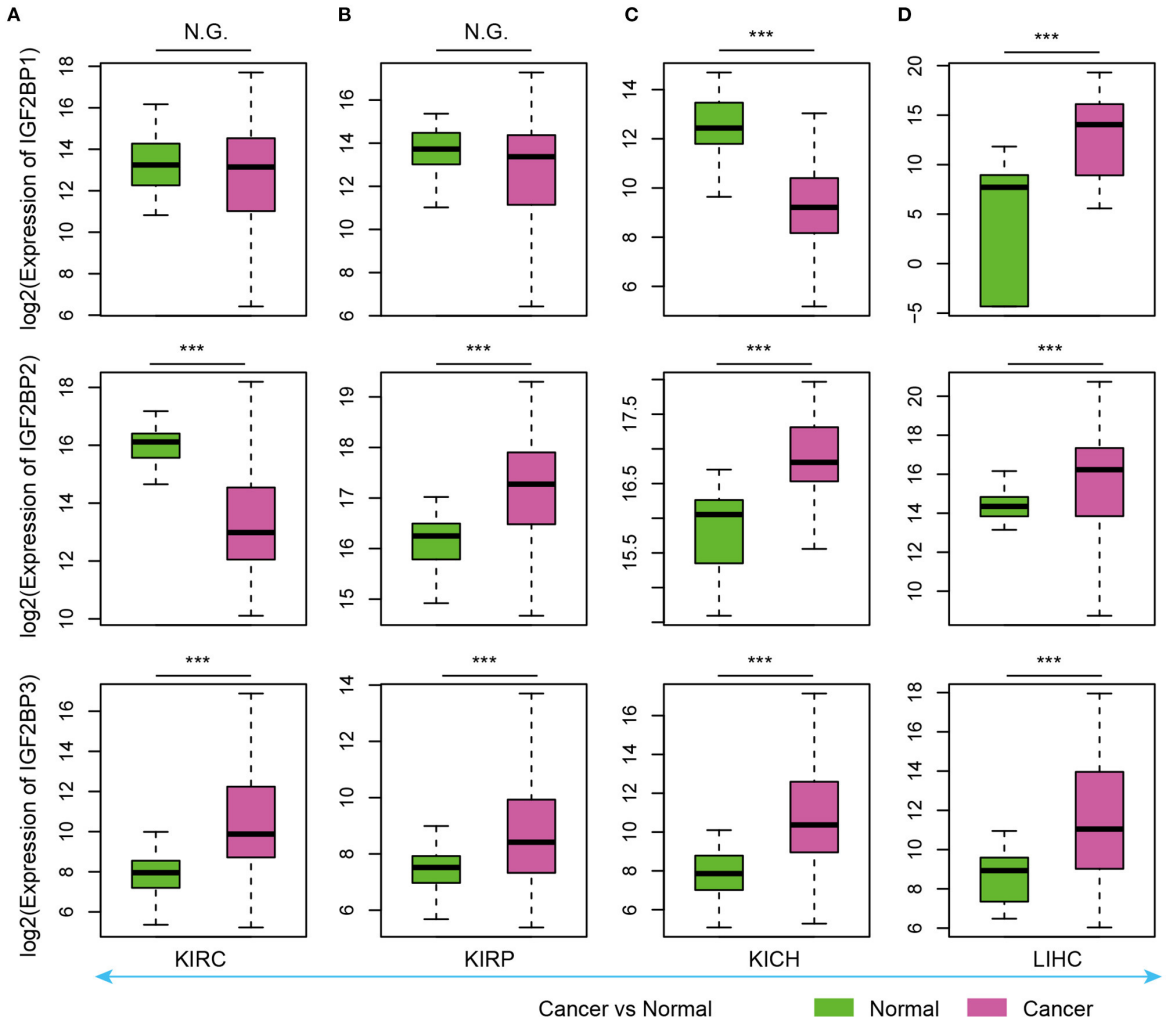
Box plots showing the expression of IGF2BP1/2/3 in cancer and normal samples. **(A)** Expression of IGF2BPs in KIRC. **(B)** Expression of IGF2BPs in KIRP. **(C)** Expression of IGF2BPs in KICH. **(D)** Expression of IGF2BPs in LIHC. ***P < 0.001 for Wilcoxon rank sum test. N.G. indicates not significant.

As evidence has shown important roles of IGF2BP1, IGF2BP2, and IGF2BP3 in cancer, we next explored their expression in various types of cancer. Here, we analyzed four cancer types with more than five normal samples. In contrast to tissue development, we found that these three genes exhibited significantly higher expression in cancer samples (Figure 3). These results suggest oncofetal reprogramming of the m^6^A regulators in cancer. This is consistent with one recent study, which revealed an oncofetal reprogramming of the tumor ecosystem in hepatocellular carcinoma (Sharma et al., 2020).

Particularly, we found that IGF2BP3 exhibited significantly higher expression in three kidney cancers and liver cancer (Figure 3). Several studies have shown that IGF2BP3 promote the stability and storage of target mRNAs, for example, MYC, and therefore affect gene expression output (Huang et al., 2020). They play critical oncogenic functions in cancer. Moreover, the depletion of IGF2BP1 had been shown to lead to an increased HULC half-life and expression, which is a long non-coding RNA highly up-regulated in liver cancer (Hammerle et al., 2013). Next, we validated the expression of IGF2BPs in another 11 cancer types. We found that IGF2BPs generally exhibited higher expression in cancer (Supplementary Figures 1–3). Taken together, these results indicated the IGF2BPs exhibited higher expression in prenatal samples and cancer patients, which might play important roles in oncofetal reprogramming in cancer.

### Clinical Associations of IGF2BP1/2/3

**Figure 4:**
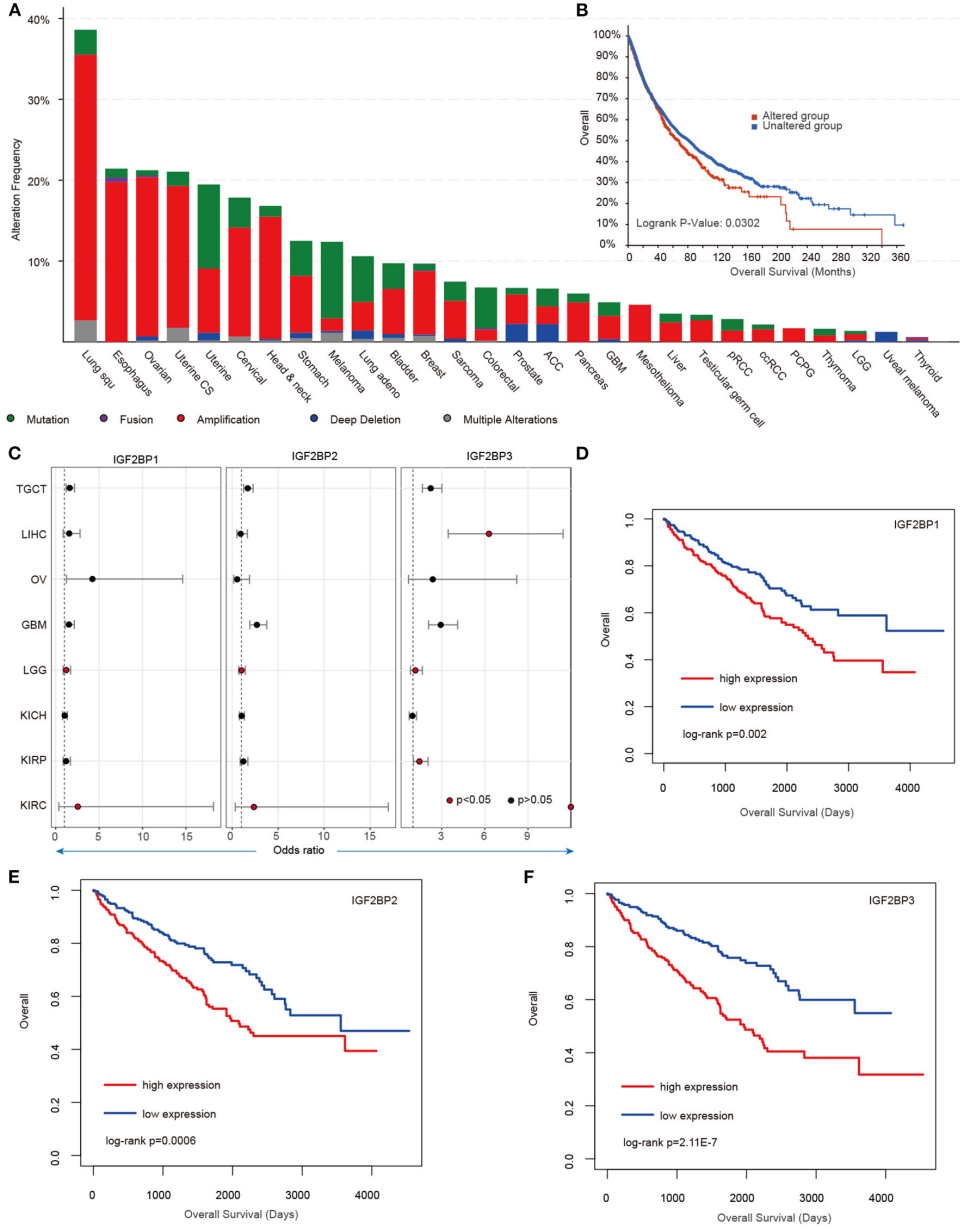
Genetic alterations and clinical association of IGF2BPs in cancer. **(A)** Bar plots showing the genetic alterations of IGF2BPs across cancer types. **(B)** Kaplan–Meier analysis of cancer patients in the IGF2BPs altered and unaltered groups. **(C)** Odds ratios of IGF2BPs across cancer types. **(D)** Kaplan–Meier analysis of KIRC cancer patients in the IGF2BP1 high expression and low expression groups. **(E)** Kaplan–Meier analysis of KIRC cancer patients in the IGF2BP2 high expression and low expression groups. **(F)** Kaplan–Meier analysis of KIRC cancer patients in the IGF2BP3 high expression and low expression groups.

Our analysis reveals higher expression of IGF2BP1/2/3 across cancer types; however, we still lack knowledge about their regulatory mechanisms in cancer. We next explored the genome alterations of IGF2BPs across cancer type. We found that there were wide genome alterations of IGF2BPs in cancer (Figure 4A). Particularly, the alteration frequencies were higher in lung cancer, ovarian cancer, and uterine cancer. Moreover, these genes exhibited higher frequency of copy number amplification than mutations. Particular, there were more mutations that occurred in the KH_1 domain in IGF2BP1. The R452C mutation exhibited the highest frequency across all TCGA cancer patients (Supplementary Figure 4). Mutually exclusive or co-occurring somatic alterations across genes suggest functional interactions in cancer (Canisius et al., 2016). We thus investigated the mutual exclusiveness and co-occurrence of m^6^A regulators in cancer. We found that the majority (179/190) of the regulator pairs were mutually exclusive or co-occurred (Supplementary Table 1), suggesting the functional cross-talk among regulators.

Next, we explored whether the genomic or transcriptome alterations of IGF2BPs were associated with clinical survival. We found that the patients with genomic mutations or copy number alterations exhibited worse survival that the unaltered patients (Figure 4B, log-rank test *p* = 0.0302). Moreover, we used the log-rank test to evaluate the association between IGF2BPs and patient survival. We found that patients with higher expression of IGF2BPs showed worse survival in multiple cancer types (Figure 4C, Supplementary Figure 5). Particularly, patients with higher expression of IGF2BPs were with higher risk in KIRC (Figures 4D–F, log-rank test *p* = 0.002, 0.0006, and 2.11E-7). All these results suggest that IGF2BPs play oncogenic roles in cancer and are potential risky prognostic factors.

### Potential Functions of IGF2BP1/2/3 in Development and Cancer

**Figure 5:**
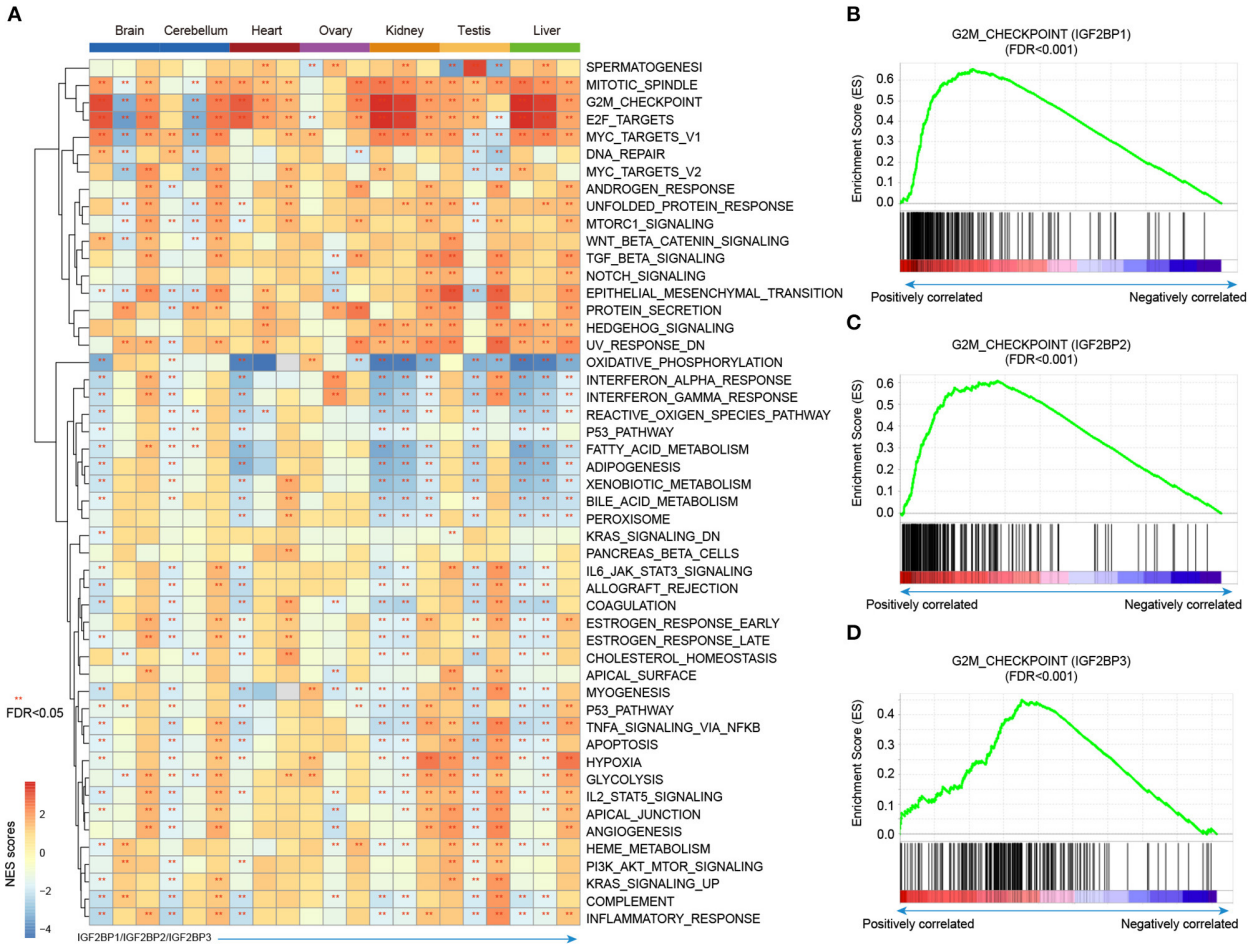
Gene set enrichment analysis of genes correlated with IGF2BPs in different tissues. **(A)** Heat map showing the normalized enrichment scores of GSEA. Functional pathways with FDR <0.05 were indicated with red stars. Each row represents one cancer hallmark-related pathway and each three column corresponding to one tissue. The first column is for IGF2BP1, second column is for IGF2BP2, and third column is for IGF2BP3. **(B)** GSEA plots showing genes correlated with IGF2BP1 in kidney development enriched in G2M checkpoint pathway. **(C)** GSEA plots showing genes correlated with IGF2BP2 in kidney development enriched in G2M checkpoint pathway. **(D)** GSEA plots showing genes correlated with IGF2BP3 in kidney development enriched in G2M checkpoint pathway. **p < 0.05 for GSEA.

Integration of transcriptome during tissue development, we revealed the dynamic expression of IGF2BPs. However, their functions are still less investigated in development and cancer. Thus, we next predicted the potential functions of IGF2BPs in development. We first calculated the correlation between the expression of IGF2BPs and other protein coding genes. Genes were ranked and subjected into GSEA analysis. Here, 50 cancer hallmark-related gene sets were considered. We found that genes positively correlated with IGF2BPs were significantly enriched in mitotic spindle, G2M, checkpoint, and E2F targets across tissues (Figure 5A and Supplementary Figures 6, 7). In addition, genes negatively correlated with IGF2BPs were significantly enriched in oxidative phosphorylation in kidney and liver tissues (Figure 5A).

Next, we explored the G2M checkpoint pathway in detail. We found that the expression of IGF2BP1 was positively correlated with MEIS1, MTF2, and EZH2, which were significantly enriched in G2M checkpoint (Figure 5B, *p* < 0.001). Moreover, the expression of IGF2BP2 was positively correlated with PAFAH1B1, CDC7, RPS6KA5, BARD1, and RBL1, which were enriched in G2M checkpoint (Figure 5C, *p* < 0.001). For IGF2BP3 regulator, the top positively correlated genes included SMAD3, ODC1, SLC7A1, and YTHDC1 (Supplementary Figures 8, 9). These genes were also enriched in G2M checkpoint pathway (Figure 5D, *p* < 0.001). We next compared the top 10 leading edges for IGF2BP1/2/3 and found that there was no overlapping gene. These results suggest that IGF2BP1/2/3 might regulate different genes in the same pathway and play important roles in tissue development and cancer.

To further understand the clinical implications of IGF2BPs in cancer, we next examined the correlation between IGF2BPs and 150 clinically actionable genes (Liberzon et al., 2015) and observed that IGF2BPs frequently interacted with actionable genes (Supplementary Figure 10). For example, IGF2BP1 interacts with PTEN, MYC, CDH1, and CTNNB1. IGF2BP2 interacts with CDKN2A, CDKN2B, and EGFR. Together, these results suggest a diverse potential of IGF2BPs in the development of novel treatment strategies.

## Discussion

The importance of m^6^A as a posttranscriptional modification in regulating RNA expression has been well-appreciated (Meyer and Jaffrey, 2014), but its global function and regulation in development remain largely unknown. This might be in part because of insufficient data of m^6^A regulation in human tissue development. Evidence has shown that the m^6^A modification is mainly determined by the expression of regulators, such as readers, writers, and erasers (Huang et al., 2018). Here, we comprehensively characterized the expression of m^6^A regulators during tissue development and cancer. We found that m^6^A regulators globally exhibited decreased expression during development in multiple tissues. However, they reversed their expression in cancer, suggesting an oncofetal reprogramming pattern. Moreover, we revealed IGF2BPs in development and cancer, providing potential therapy targets for cancer.

Given that m^6^A regulators exhibited widespread perturbations in expression, we explored the potential mechanism for regulating their expression. We found that IGF2BPs were with higher frequency of genomic alterations, particular copy number amplification across cancer types. This is consistent with one of recent studies (Li et al., 2019b). Although we revealed the expression perturbation of m^6^A regulators during tissue development and cancer, we cannot evaluate their direct function in downstream targets. This is mainly because we still lack paired m^6^A modification data in tissue development. Xia et al. had generated 21 whole transcriptome m^6^A methylomes across major fetal tissues (Xiao et al., 2019); however, no adult tissues were included. Moreover, Zhang et al. generated 27 m^6^A methylomes across major adult tissues (Zhang et al., 2020a). Besides of protein coding genes, noncoding RNAs have been found to play important roles in development and cancer (Li et al., 2020; Xu et al., 2020a; Zhang et al., 2020b). Integration of these data in future will provide novel insights into the roles of m^6^A regulators in development and cancer.

Moreover, we found that IGF2BPs might regulate the same pathway through different genes, suggesting their cooperative regulation. Cooperative regulation has been revealed for number of regulators, such as enhancer (Chen et al., 2019a), miRNA (Xu et al., 2011; Shao et al., 2019), and RBPs (Li et al., 2019a). Investigation of the cooperative regulation between m^6^A regulators will identify novel regulatory modules that play important roles in tissue development and cancer. For example, Rao et al. have revealed that cross-talk among writers, readers, and erasers can regulate cancer growth and progression (Panneerdoss et al., 2018). Despite these recent developments, much effort needs to be done to understand the downstream targets of the altered m^6^A regulators. With the increase of m^6^A methylome and functional datasets, we will get much more insights into the cooperative regulation among regulators, as well as their regulation to RNA methylation.

In summary, our comprehensive analysis of the dynamic expression of m^6^A regulators during human tissue development and cancer reveals their important roles. Particularly, IGF2BPs exhibited decreased expression in tissue development and reversed expression in cancer. Expression perturbation of IGF2BPs was correlated with cancer-related functions and associated with clinical survival in cancer.

## Data Availability Statement

The original contributions presented in the study are included in the article/Supplementary Material, further inquiries can be directed to the corresponding author/s.

## Author Contributions

YL, JX, and XL conceived, designed, and supervised the study. YZ and SX collected the data and performed all data analysis. GX, YG, SL, KZ, ZT, and JG help plotting the figures and data analysis. YL and JX drafted the manuscript. All authors reviewed and approved the final manuscript.

## Conflict of Interest

The authors declare that the research was conducted in the absence of any commercial or financial relationships that could be construed as a potential conflict of interest.

## References

[B1] Bin Lim S. Chua M. L. K. Yeong J. P. S. Tan S. J. Lim W. T. Lim C. T. (2019). Pan-cancer analysis connects tumor matrisome to immune response. NPJ Precis. Oncol. 3, 15 10.1038/s41698-019-0087-0 31123708 PMC6531473

[B2] Cancer Genome Atlas Research N. Weinstein J. N. Collisson E. A. Mills G. B. Shaw K. R. Ozenberger B. A.. (2013). The Cancer Genome Atlas Pan-Cancer analysis project. Nat. Genet. 45, 1113–1120. 10.1038/ng.2764 24071849 PMC3919969

[B3] Canisius S. Martens J. W. Wessels L. F. (2016). A novel independence test for somatic alterations in cancer shows that biology drives mutual exclusivity but chance explains most co-occurrence. Genome Biol. 17:261. 10.1186/s13059-016-1114-x 27986087 PMC5162102

[B4] Cardoso-Moreira M. Halbert J. Valloton D. Velten B. Chen C. Shao Y. (2019). Gene expression across mammalian organ development. Nature 571, 505–509. 10.1038/s41586-019-1338-5 31243369 PMC6658352

[B5] Chen H. Gao S. Liu W. Wong C. C. Wu J. Wu J.. (2020). RNA m(6)A methyltransferase METTL3 facilitates colorectal cancer by activating m(6)A-GLUT1-mTORC1 axis and is a therapeutic target. Gastroenterology. 10.1053/j.gastro.2020.11.013. [Epub ahead of print]. 33217448

[B6] Chen H. Xiao J. Shao T. Wang L. Bai J. Lin X.. (2019a). Landscape of enhancer-enhancer cooperative regulation during human cardiac commitment. Mol. Ther. Nucleic. Acids 17, 840–851. 10.1016/j.omtn.2019.07.015 31465963 PMC6717080

[B7] Chen Y. Peng C. Chen J. Chen D. Yang B. He B.. (2019b). WTAP facilitates progression of hepatocellular carcinoma via m6A-HuR-dependent epigenetic silencing of ETS1. Mol. Cancer 18:127. 10.1186/s12943-019-1053-8 31438961 PMC6704583

[B8] de Vasconcellos J. F. Tumburu L. Byrnes C. Lee Y. T. Xu P. C. Li M.. (2017). IGF2BP1 overexpression causes fetal-like hemoglobin expression patterns in cultured human adult erythroblasts. Proc. Natl. Acad. Sci. U.S.A. 114, E5664–E5672. 10.1073/pnas.1609552114 28652347 PMC5514697

[B9] Dominissini D. Moshitch-Moshkovitz S. Schwartz S. Salmon-Divon M. Ungar L. Osenberg S.. (2012). Topology of the human and mouse m6A RNA methylomes revealed by m6A-seq. Nature 485, 201–206. 10.1038/nature11112 22575960

[B10] Gao J. Aksoy B. A. Dogrusoz U. Dresdner G. Gross B. Sumer S. O.. (2013). Integrative analysis of complex cancer genomics and clinical profiles using the cBioPortal. Sci. Signal 6:pl1. 10.1126/scisignal.2004088 23550210 PMC4160307

[B11] Hammerle M. Gutschner T. Uckelmann H. Ozgur S. Fiskin E. Gross M.. (2013). Posttranscriptional destabilization of the liver-specific long noncoding RNA HULC by the IGF2 mRNA-binding protein 1 (IGF2BP1). Hepatology 58, 1703–1712. 10.1002/hep.26537 23728852

[B12] Huang H. Weng H. Sun W. Qin X. Shi H. Wu H. (2018). Recognition of RNA N(6)-methyladenosine by IGF2BP proteins enhances mRNA stability and translation. Nat. Cell Biol. 20, 285–295. 10.1038/s41556-018-0045-z 29476152 PMC5826585

[B13] Huang H. Weng H. Sun W. Qin X. Shi H. Wu H.. (2020). Publisher correction: recognition of RNA N(6)-methyladenosine by IGF2BP proteins enhances mRNA stability and translation. Nat. Cell Biol. 22:1288. 10.1038/s41556-020-00580-y 32855523

[B14] Leek J. T. Johnson W. E. Parker H. S. Jaffe A. E. Storey J. D. (2012). The sva package for removing batch effects and other unwanted variation in high-throughput experiments. Bioinformatics 28, 882–883. 10.1093/bioinformatics/bts034 22257669 PMC3307112

[B15] Li T. Hu P. S. Zuo Z. Lin J. F. Li X. Wu Q. N.. (2019). METTL3 facilitates tumor progression via an m(6)A-IGF2BP2-dependent mechanism in colorectal carcinoma. Mol. Cancer 18:112. 10.1186/s12943-019-1038-7 31230592 PMC6589893

[B16] Li Y. Jiang T. Zhou W. Li J. Li X. Wang Q.. (2020). Pan-cancer characterization of immune-related lncRNAs identifies potential oncogenic biomarkers. Nat. Commun. 11:1000. 10.1038/s41467-020-14802-2 32081859 PMC7035327

[B17] Li Y. McGrail D. J. Xu J. Li J. Liu N. N. Sun M.. (2019a). MERIT: systematic analysis and characterization of mutational effect on RNA interactome topology. Hepatology 70, 532–546. 10.1002/hep.30242 30153342 PMC6538468

[B18] Li Y. Xiao J. Bai J. Tian Y. Qu Y. Chen X.. (2019b). Molecular characterization and clinical relevance of m(6)A regulators across 33 cancer types. Mol. Cancer 18:137. 10.1186/s12943-019-1066-3 31521193 PMC6744659

[B19] Liberzon A. Birger C. Thorvaldsdottir H. Ghandi M. Mesirov J. P. Tamayo P. (2015). The Molecular Signatures Database (MSigDB) hallmark gene set collection. Cell Syst. 1, 417–425. 10.1016/j.cels.2015.12.004 26771021 PMC4707969

[B20] Liberzon A. Subramanian A. Pinchback R. Thorvaldsdottir H. Tamayo P. Mesirov J. P. (2011). Molecular signatures database (MSigDB) 3.0. Bioinformatics 27, 1739–1740. 10.1093/bioinformatics/btr260 21546393 PMC3106198

[B21] Liu J. Yue Y. Han D. Wang X. Fu Y. Zhang L.. (2014). A METTL3-METTL14 complex mediates mammalian nuclear RNA N6-adenosine methylation. Nat. Chem. Biol. 10, 93–95. 10.1038/nchembio.1432 24316715 PMC3911877

[B22] Liu X. Qin J. Gao T. Li C. He B. Pan B.. (2020). YTHDF1 facilitates the progression of hepatocellular carcinoma by promoting FZD5 mRNA translation in an m6A-dependent manner. Mol. Ther. Nucleic Acids 22, 750–765. 10.1016/j.omtn.2020.09.036 33230473 PMC7595883

[B23] Meyer K. D. Jaffrey S. R. (2014). The dynamic epitranscriptome: N6-methyladenosine and gene expression control. Nat. Rev. Mol. Cell Biol. 15, 313–326. 10.1038/nrm3785 24713629 PMC4393108

[B24] Meyer K. D. Saletore Y. Zumbo P. Elemento O. Mason C. E. Jaffrey S. R. (2012). Comprehensive analysis of mRNA methylation reveals enrichment in 3' UTRs and near stop codons. Cell 149, 1635–1646. 10.1016/j.cell.2012.05.003 22608085 PMC3383396

[B25] Mootha V. K. Lindgren C. M. Eriksson K. F. Subramanian A. Sihag S. Lehar J.. (2003). PGC-1alpha-responsive genes involved in oxidative phosphorylation are coordinately downregulated in human diabetes. Nat. Genet 34, 267–273. 10.1038/ng1180 12808457

[B26] Panneerdoss S. Eedunuri V. K. Yadav P. Timilsina S. Rajamanickam S. Viswanadhapalli S.. (2018). Cross-talk among writers, readers, and erasers of m(6)A regulates cancer growth and progression. Sci. Adv. 4:eaar8263. 10.1126/sciadv.aar8263 30306128 PMC6170038

[B27] Shao T. Wang G. Chen H. Xie Y. Jin X. Bai J.. (2019). Survey of miRNA-miRNA cooperative regulation principles across cancer types. Brief. Bioinformatics 20, 1621–1638. 10.1093/bib/bby038 29800060

[B28] Sharma A. Seow J. J. W. Dutertre C. A. Pai R. Bleriot C. Mishra A.. (2020). Onco-fetal reprogramming of endothelial cells drives immunosuppressive macrophages in hepatocellular carcinoma. Cell 183, 377-394 e321. 10.1016/j.cell.2020.08.040 32976798

[B29] Subramanian A. Tamayo P. Mootha V. K. Mukherjee S. Ebert B. L. Gillette M. A.. (2005). Gene set enrichment analysis: a knowledge-based approach for interpreting genome-wide expression profiles. Proc. Natl. Acad. Sci. U.S.A. 102, 15545–15550. 10.1073/pnas.0506580102 16199517 PMC1239896

[B30] Wickham H. (2009). ggplot2: Elegant Graphics for Data Analysis. Springer 10.1007/978-0-387-98141-3

[B31] Xiao S. Cao S. Huang Q. Xia L. Deng M. Yang M.. (2019). The RNA N(6)-methyladenosine modification landscape of human fetal tissues. Nat. Cell Biol. 21, 651–661. 10.1038/s41556-019-0315-4 31036937

[B32] Xu J. Li C. X. Li Y. S. Lv J. Y. Ma Y. Shao T. T.. (2011). MiRNA-miRNA synergistic network: construction via co-regulating functional modules and disease miRNA topological features. Nucleic Acids Res. 39, 825–836. 10.1093/nar/gkq832 20929877 PMC3035454

[B33] Xu J. Shao T. Song M. Xie Y. Zhou J. Yin J.. (2020a). MIR22HG acts as a tumor suppressor via TGFbeta/SMAD signaling and facilitates immunotherapy in colorectal cancer. Mol. Cancer 19:51. 10.1186/s12943-020-01174-w 32127004 PMC7055097

[B34] Xu S. Tang L. Dai G. Luo C. Liu Z. (2020b). Expression of m6A regulators correlated with immune microenvironment predicts therapeutic efficacy and prognosis in gliomas. Front Cell Dev. Biol. 8:594112. 10.3389/fcell.2020.594112 33240891 PMC7683617

[B35] Zhang H. Shi X. Huang T. Zhao X. Chen W. Gu N.. (2020a). Dynamic landscape and evolution of m6A methylation in human. Nucleic Acids Res. 48, 6251–6264. 10.1093/nar/gkaa347 32406913 PMC7293016

[B36] Zhang J. Li S. Zhang L. Xu J. Song M. Shao T.. (2020b). RBP EIF2S2 promotes tumorigenesis and progression by regulating MYC-mediated inhibition via FHIT-related enhancers. Mol. Ther. 28, 1105–1118. 10.1016/j.ymthe.2020.02.004 32059763 PMC7132626

