# Supplementary material for: Dynamic Expression of m6A Regulators During Multiple Human Tissue Development and Cancers
Source: Front Cell Dev Biol. 2021 Jan 26;8:629030. doi: 10.3389/fcell.2020.629030 (PMC7870680; doi:10.3389/fcell.2020.629030)
Supplement: Supplementary file 3 [file Data_Sheet_1.PDF]

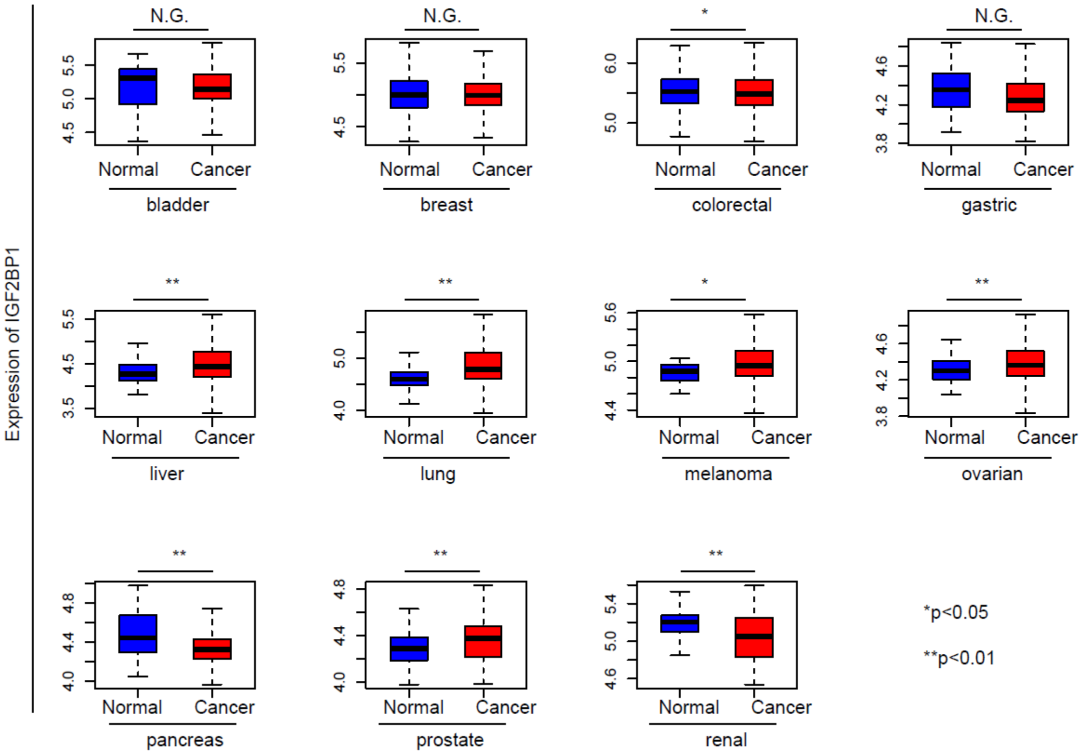

Figure S1. Expressions of IGF2BP1 across cancer types. Blue, normal samples; red, cancer samples.

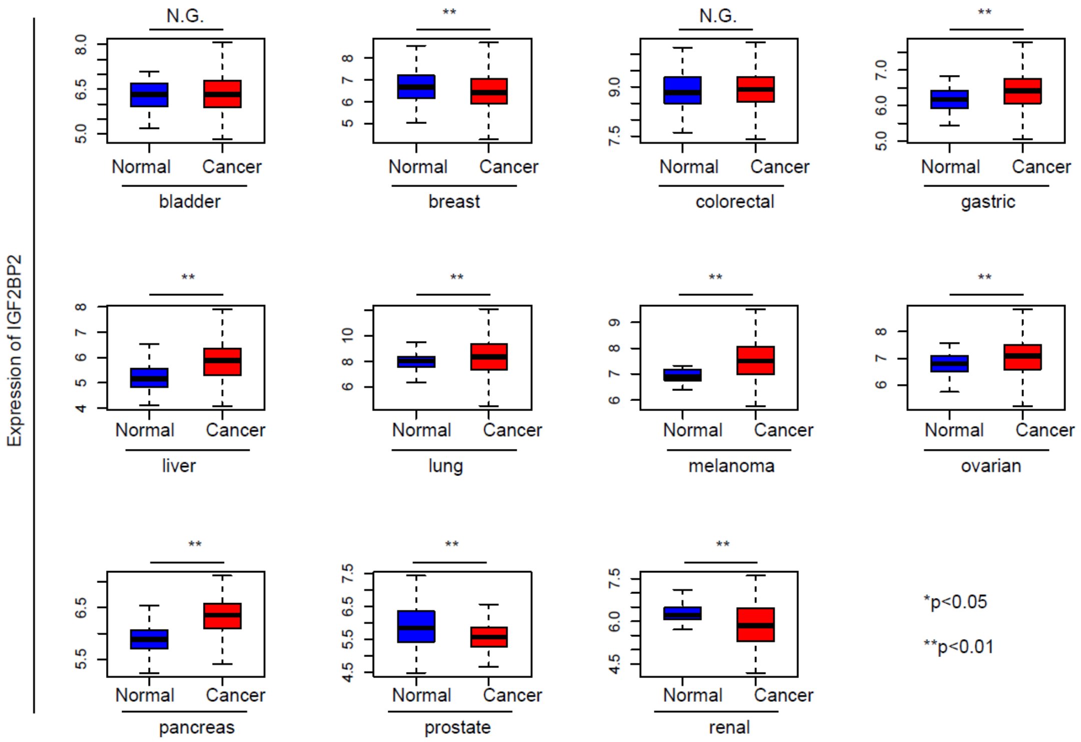

Figure S2. Expressions of IGF2BP2 across cancer types. Blue, normal samples; red, cancer samples.

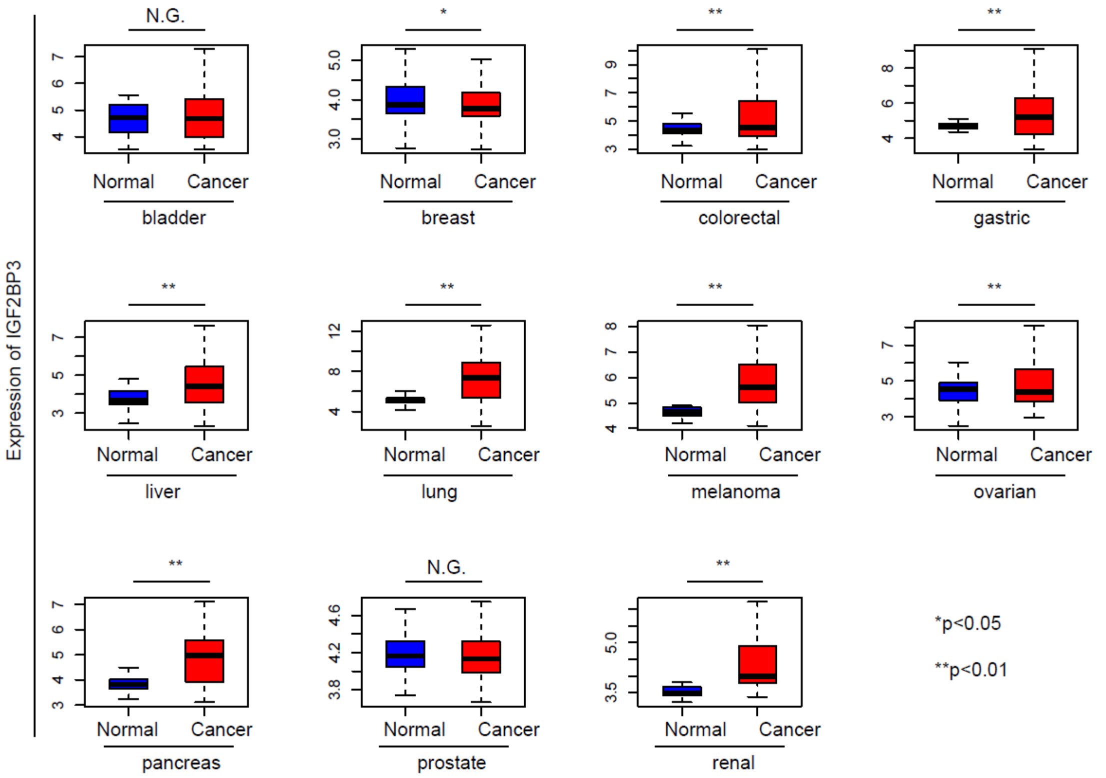

Figure S3. Expressions of IGF2BP3 across cancer types. Blue, normal samples; red, cancer samples.

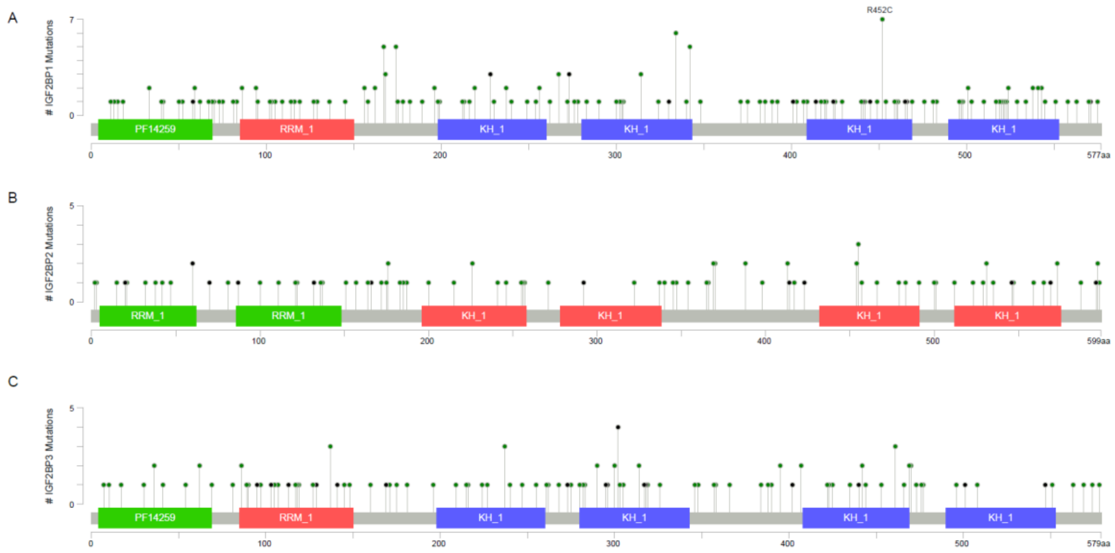

Figure S4. Mutations of IGF2BPs across cancer types. A for IGF2BP1; B for IGF2BP2 and C for IGF2BP3.

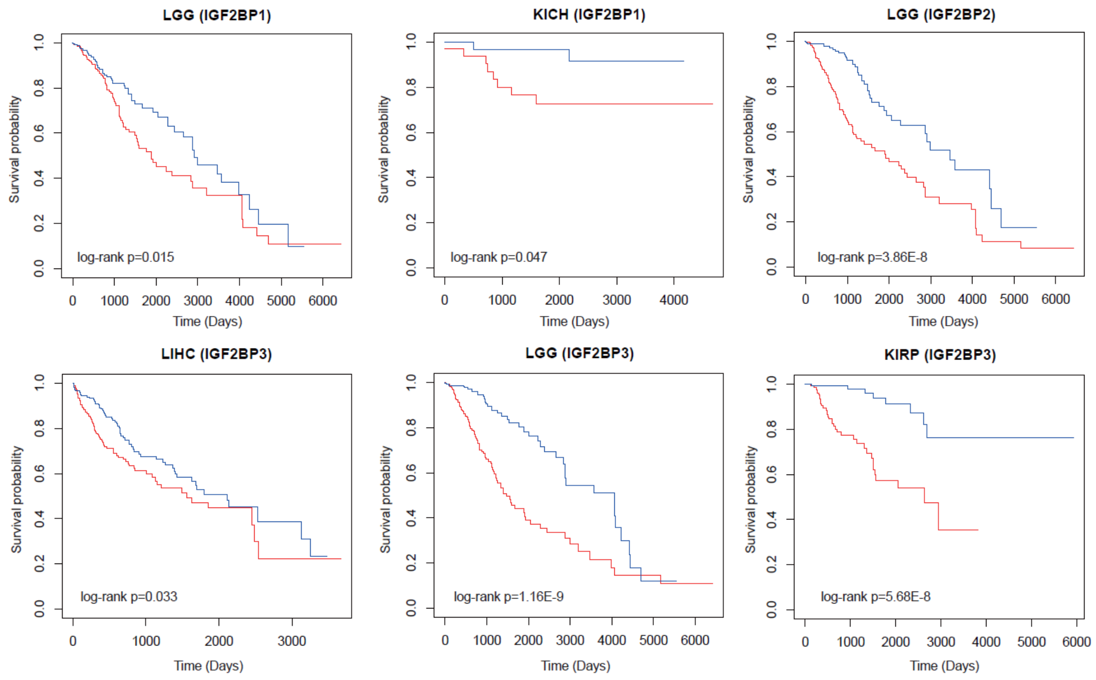

Figure S5. Kaplan–Meier analysis of cancer patients in the IGF2BPs high expression and low expression groups.

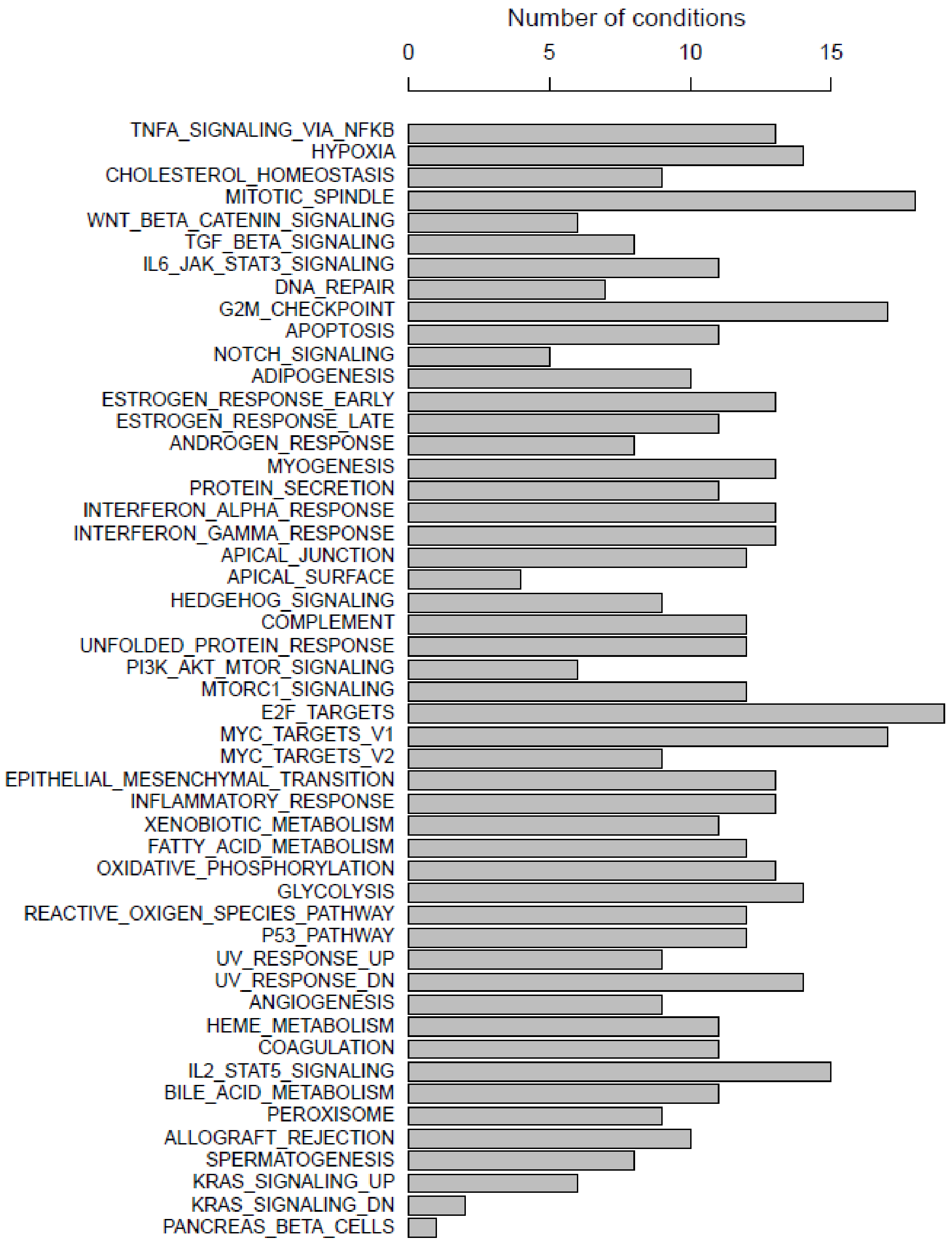

Figure S6. Number of conditions for each hallmark-related pathway.

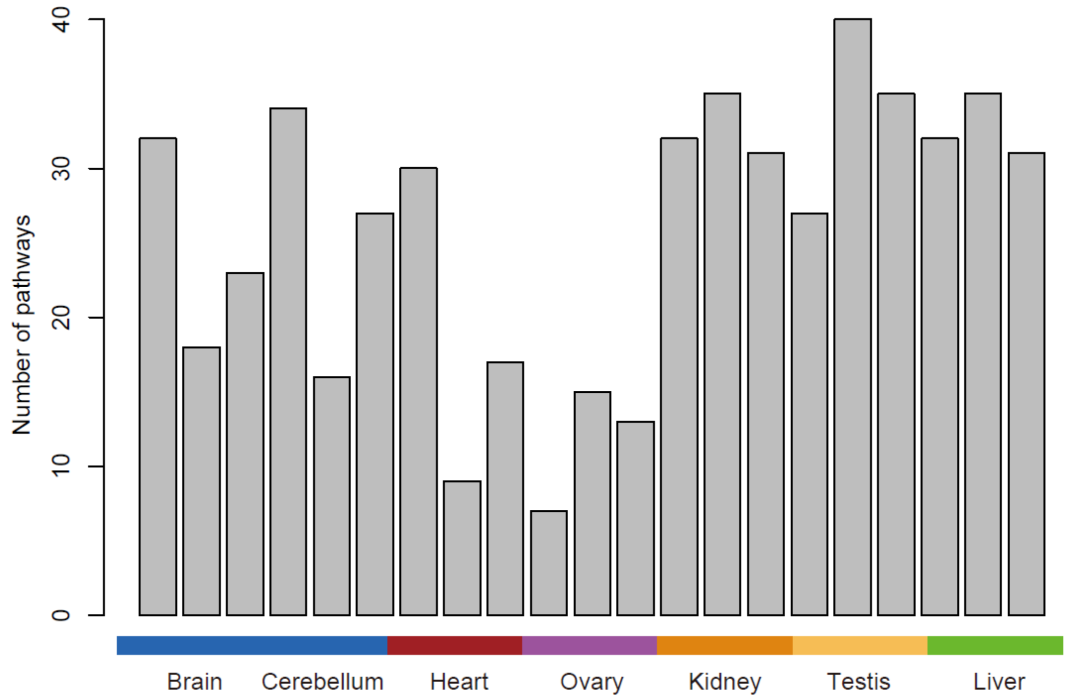

Figure S7. Number of pathways enriched by genes correlated with IGF2BPs.

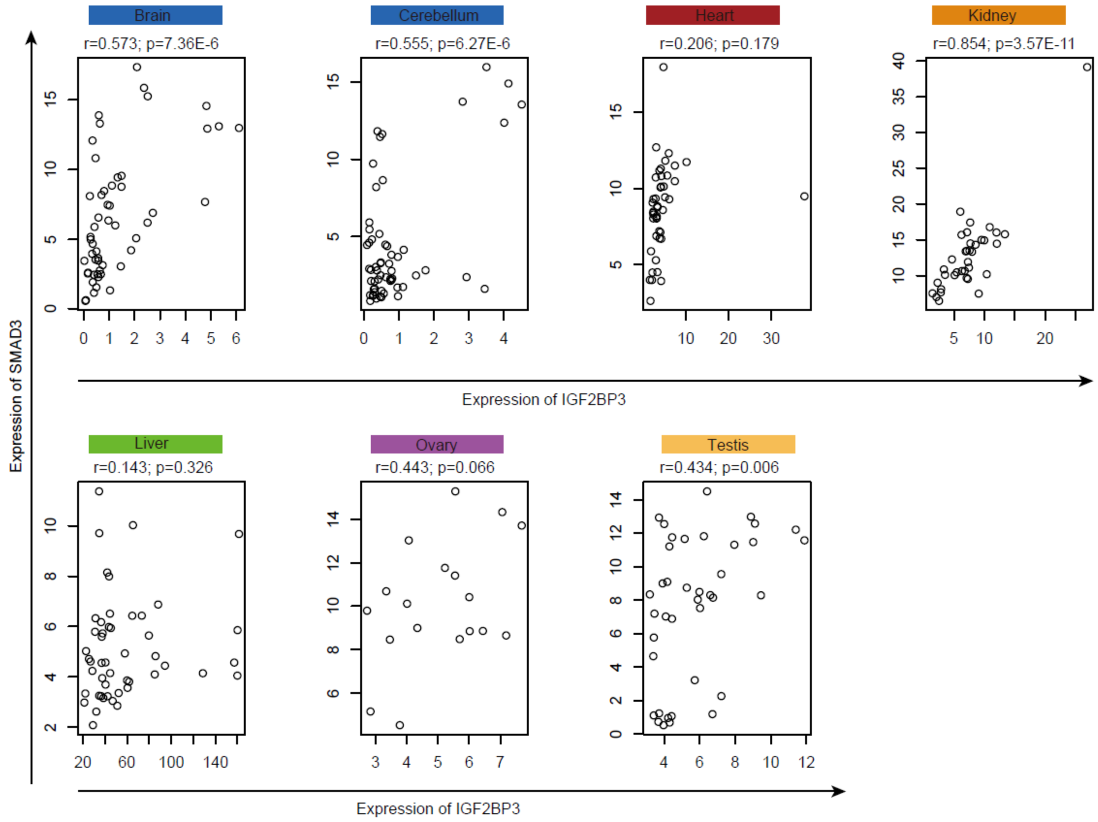

Figure S8. Scatter plots showing the correlation between expression of IGF2BP3 and SMAD3.

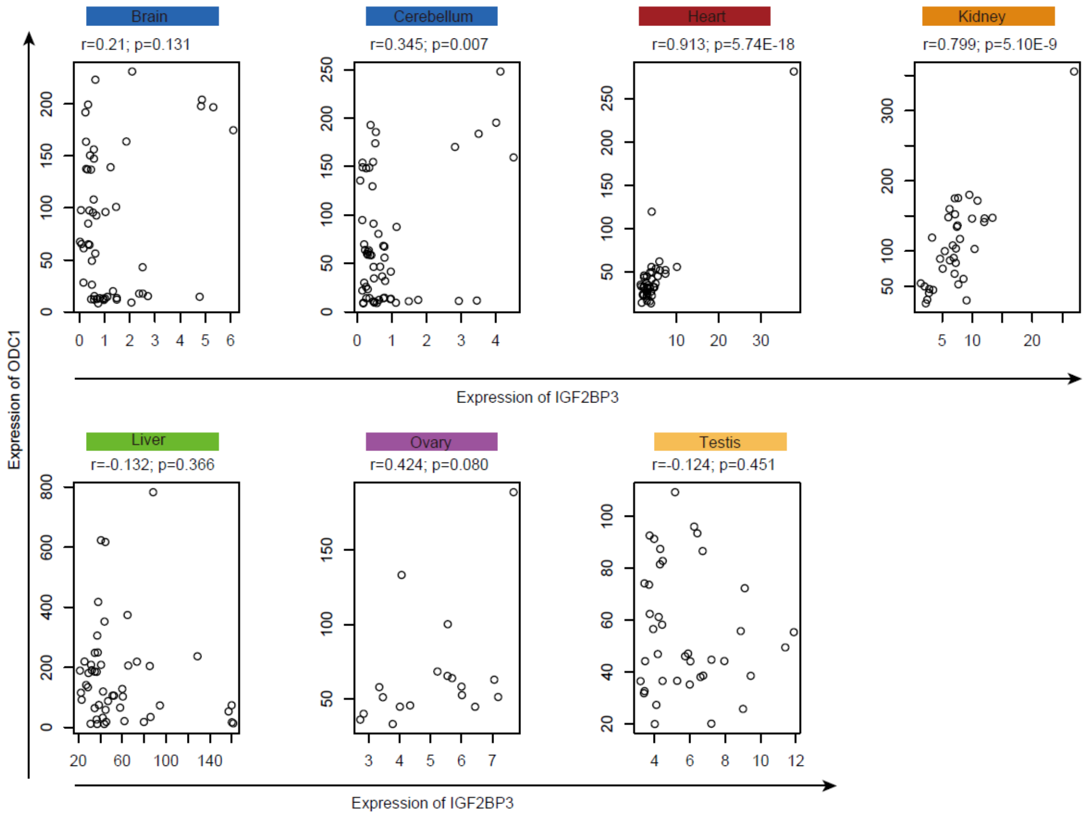

Figure S9. Scatter plots showing the correlation between expression of IGF2BP3 and ODC1.

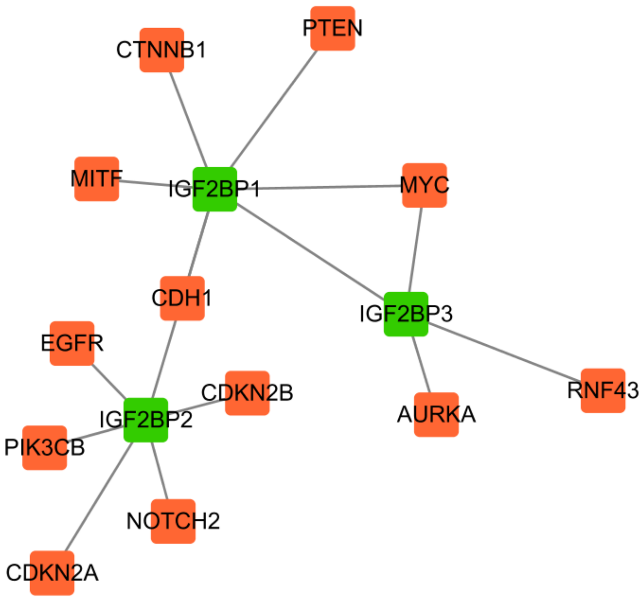

Figure S10. Protein interactions among IGF2BPs and clinically actionable genes.
